# Therapy-Related Acute Myeloid Leukemia Following TCHP Chemotherapy in Two HER2+ Breast Cancer Patients

**DOI:** 10.7759/cureus.11957

**Published:** 2020-12-07

**Authors:** Navroop Gill, Anjana Chandran, Brian Adley, Jacob Bitran

**Affiliations:** 1 Internal Medicine, Chicago Medical School, Rosalind Franklin University of Medicine, North Chicago, USA; 2 Internal Medicine, Advocate Lutheran General Hospital, Park Ridge, USA; 3 Pathology, Advocate Lutheran General Hospital, Park Ridge, USA; 4 Oncology, Advocate Lutheran General Hospital, Park Ridge, USA

**Keywords:** breast cancer, breast cancer outcomes, t-aml, t-mds, tchp, docetaxel, carboplatin, pertuzumab, trastuzumab

## Abstract

Increased risk for the development of therapy-induced myeloid leukemia following the treatment of breast cancer has typically been associated with the use of regimens containing anthracyclines or alkylating agents. We present two cases of estrogen receptor-positive/progesterone receptor-positive/human epidermal growth factor receptor 2-positive (ER+/PR+/HER2+) breast cancer patients, treated with a non-anthracycline, non-alkylating regimen of trastuzumab, carboplatin, docetaxel, and pertuzumab (TCHP), who developed therapy-related acute myeloid leukemia (t-AML) within 30 months of the completion of treatment. Both patients had marked cytogenetic abnormalities, including deletions of chromosomes 5 and 7, and highly aggressive disease that resulted in a poor prognosis. While platinum and taxane-based chemotherapy regimens have been previously linked to the development of t-AML or therapy-related myelodysplastic syndrome (t-MDS) following treatment for ovarian cancer, they have not yet been shown to increase the risk of t-AML/t-MDS after their use for breast cancer therapy. As TCHP is widely used for the treatment of HER2/neu overexpressed breast cancer, these cases highlight the need to further evaluate the link between taxane and platinum-based chemotherapeutics for breast cancer and the development of t-AML/t-MDS.

## Introduction

Breast cancer incidence in the United States has continued to increase with an estimated 12.9% of women being diagnosed with breast cancer during their lifetime. Approximately 66% of newly diagnosed breast cancer patients will be treated with chemotherapy, which has been associated with a 1.5 to 4-fold increased risk of developing therapy-related myelodysplastic syndrome (t-MDS) [1-2]. The risk for the development of treatment-related acute myeloid leukemia (t-AML) is higher than that for t-MDS and higher in those receiving chemotherapy for regional or distant-stage disease as opposed to early-stage disease [2]. Herein, we report two cases of t-AML following docetaxel, carboplatin, trastuzumab, and pertuzumab (TCHP) therapy for the treatment of human epidermal growth factor receptor 2-positive (HER2+) breast cancer.

## Case presentation

Case 1

**Figure 1:**
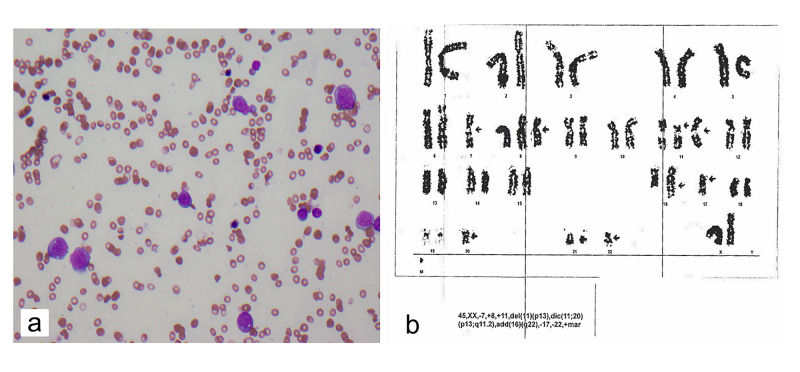
Bone Marrow Biopsy and Karyotype of Case 1 (a) bone marrow aspirate showing blast cells consistent with acute myeloid leukemia (AML); (b) karyotype of leukemic cells within the bone marrow

In November 2017, a 58-year-old woman was found to have Stage IIIB, Grade 3 lobular carcinoma that was estrogen receptor-positive (ER+), progesterone receptor-positive (PR+), and HER2/neu amplified with right axillary lymph nodes that were positive for breast cancer. She completed six cycles of neoadjuvant TCHP with clinical complete response (CR) and then underwent a modified mastectomy in April 2018. She completed radiation therapy to her right chest wall and axilla in July 2018. After completing her one year of trastuzumab, she started adjuvant endocrine therapy with letrozole. She was followed closely in the clinic. In August 2020, 33 months after the original diagnosis and 30 months after completion of neoadjuvant chemotherapy, she was found to have pancytopenia. A bone marrow biopsy was performed and confirmed AML (Figure 1A) with complex karyotype showing deletion of 5q31, monosomy 7, loss of CEP7, trisomy 8, and deletion of 20q12 (Figure 1B). Shortly after admission, the patient died from invasive fungal pneumonia. 

Case 2

**Figure 2:**
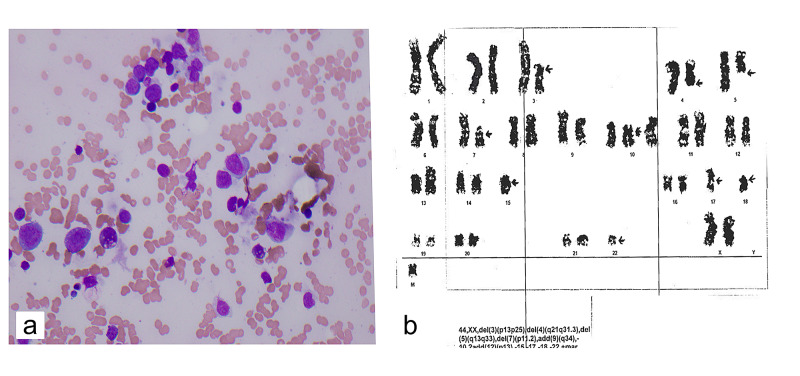
Bone Marrow Biopsy and Karyotype of Case 2 (a) bone marrow aspirate showing blast cells consistent with acute myeloid leukemia (AML); (b) karyotype of leukemic cells within the bone marrow

In September 2017, a 69-year-old female was diagnosed with Stage IIIA, Grade 2 invasive ductal carcinoma that was ER+, PR+, and HER2/neu amplified. She then underwent bilateral mastectomies with reconstruction. At the time of surgery in November 2017, she was found to have a 1.6 cm focus of invasive carcinoma and five of 23 positive lymph nodes. She then completed six cycles of TCHP. She completed radiation, as well as one year of trastuzumab, and was started on anastrozole in September 2018. She was followed closely, and in January 2020 was found to have pancytopenia 29 months from the original diagnosis and 20 months following the completion of chemotherapy. A bone marrow biopsy revealed AML (20% - 30% blasts) (Figure 2A) with complex cytogenetics most consistent with t-AML which included deletions of 3p, 4q, and 5q, an unbalanced rearrangement involving chromosomes 7 and 22 resulting in the loss of 7p, additional material of unknown origin on 9q, and loss of chromosomes 17 and 22 (Figure 2B). The patient was admitted to the hospital and started on chemotherapy for t-AML with daunorubicin-cytarabine. She failed induction and re-induction. In late March 2020, she was started on salvage decitabine and venetoclax. Her course was complicated by neutropenic fever and hemophagocytic lymphohistiocytosis. Due to the worsening of her overall clinical condition, the patient and her family chose to pursue hospice care and she died in late April 2020.

## Discussion

The link between t-AML/t-MDS following platinum and taxane-based chemotherapy has been established in patients treated for ovarian cancer [2-4]. To our knowledge, there is only one case report that has reported the causal link of TCHP in a patient with breast cancer who subsequently developed t-AML [5]. In patients treated for ovarian cancer, platinum-containing compounds result in an increased risk of t-MDS/t-AML, with carboplatin specifically, resulting in a latency period of approximately four years and more rare AML subtypes [3-4]. Studies have correlated an increased relative risk of t-MDS/t-AML with increasing doses and cycles of platinum-based therapy. This is consistent with the known leukemogenicity of platinum compounds, which induce DNA interstrand crosslinks [1, 3, 6]. Case reports investigating taxane-related t-AML following gynecological malignancies had a mean onset time of approximately nine months from the beginning of chemotherapy to the diagnosis of leukemia. These case studies specifically included paclitaxel and found no dose-related increase in risk but rather that patients developing t-AML simply had an idiosyncratic response to treatment [4]. Commonly, secondary AML due to paclitaxel-containing chemotherapy results in the M4 subtype with cytogenetic abnormalities and the absence of an antecedent hematologic disorder [4, 7]. 

The link between alkylating and anthracycline agents and t-MDS in breast cancer has been well-established and studied extensively [2, 8-11]. Disease related to alkylating agents typically presents as t-MDS five to seven years following initial treatment. Two-thirds of these patients usually present with bone marrow failure characterized by pancytopenia and typical myelodysplastic marrow changes, including myeloblasts with megaloblastic changes in cell lines [3-4]. Typical cytogenetic changes noted on marrow analysis include the deletion of chromosomes 5 and 7 which confer a poor prognosis and unfavorable response to treatment [4, 6]. t-AML resulting from prior treatment with either topoisomerase II or anthracycline-based chemotherapy regimens typically develops within five years post-chemotherapy, with a mean of two to three years post-treatment. It has no association with t-MDS and is commonly defined by the 11q23 cytogenetic abnormality [10-11]. Studies have suggested that non-anthracycline containing regimens have a decreased incidence of leukemia, as compared to anthracycline-containing regimens when used in the treatment for breast cancer [12]. Additionally, the monoclonal antibody trastuzumab used for the treatment of HER2/neu breast cancer currently has no known association with the development of t-AML or t-MDS [12]. 

The exact pathophysiology underlying the development of t-AML has not yet been identified. It has been proposed that generalized genomic instability induced by chemotherapy may be a critical factor in the origin of genetic alterations that drive the disease. In a case study of t-AML following treatment for breast cancer, several chromosomal abnormalities were noted, including MYC, KMT2A, TP53 which were impacted by rearrangement, deletion, or amplification that were thought to drive disease progression [5]. The MYC amplification and overexpression promoted increased proliferation, instability, and poor outcome. Chromosome 11 contained a 11q23.3 rearrangement affecting KMT2A which has previously been linked to leukemogenesis, and there was a loss of function of TP53 on chromosome 17 that also likely contributed to the development of the t-AML [5]. In studies looking at the development of t-AML after any malignancy and chemotherapy regimen, TP53 was reported to be mutated in 20% - 50% of patients with t-AML, which is significantly higher than patients with *de novo* AML. Harboring this mutation is linked to intrinsic therapy resistance and worse overall prognosis. Differences in cytogenetics between t-AML in comparison with *de novo* AML also included a higher incidence of mutations in PTPN11 and a lower incidence of FLT3 and NPM1 [13]. 

Typically, the diagnosis of t-MDS/t-AML is made via a combination of blood counts, bone marrow aspirates, flow cell cytometry, and karyotype analysis [3, 5]. The treatment for t-AML is tailored to the patient based upon age, comorbidities, performance status, and cytogenetic features. Patients with t-AML have an unfavorable prognosis with a median survival of eight to 10 months after diagnosis [10-11, 13]. The only curative therapy is allogeneic bone marrow transplantation following induction chemotherapy with daunorubicin-cytarabine (CPX-351) [13].

## Conclusions

Herein, we presented the cases of two patients who developed t-AML 20 to 30 months following the completion of primary TCHP chemotherapy for ER+, PR+, HER2+ breast cancer. The use of taxane and platinum-based regimens for the initial treatment of breast cancer has not previously been linked to an increased risk for the development of t-AML. While this link has been established following treatment for ovarian cancer, it warrants further exploration in patients treated for primary breast cancer. Furthermore, the complex cytogenetics of the presented patients are consistent with previous reports of t-AML, conferring an aggressive clinical course and poor overall prognosis. Our case studies add to the limited body of literature linking TCHP chemotherapy for breast cancer to the development of t-AML. Further investigation into this association is warranted, given its widespread use for the treatment of breast cancer.
